# Computational Investigation into the Interactions of Traditional Chinese Medicine Molecules of WenQingYin with GluR2

**DOI:** 10.3390/ijms18071443

**Published:** 2017-07-05

**Authors:** Yu-Hui Tseng, Po-Hsiang Chuang, Yu-Ren Huang, Cheng-Lung Chen

**Affiliations:** 1 Department of Chemistry, National Sun Yat-Sen University, 80424 Kaohsiung, Taiwan; tsenglittleyuyu@gmail.com (Y.-H.T.); calcium7@hotmail.com (P.-H.C.); g971101@yahoo.com.tw (Y.-R.H.); 2 Department of Applied Science, R.O.C. Naval Academy, 81345 Kaohsiung, Taiwan

**Keywords:** molecular docking, molecular dynamics simulation, glutamate receptor 2, traditional Chinese medicine

## Abstract

Docking and molecular dynamics simulations have been carried out to investigate the interaction of a traditional Chinese medicine, WenQingYin, with the glutamate receptor 2 (GluR2) subunit of the α-amino-3-hydroxy-5-methyl-4-isoxazolepropionic acid (AMPA) receptor. Four representative drug components of WenQingYin, namely 2-(3,4-dihydroxyphenyl)-5,6,7-trihydroxy-4*H*-chromen-4-one (PHF), 4-hydroxy-3-methoxybenzoic acid (HMB), 4-(2,3-dihydroxy-3-methylbutoxy)-7*H*-furo[3,2-g]chromen-7-one (DHMBP) and methyl 7-formylcyclopenta[*c*]pyran-4-carboxylate (cerbinal), and their complexes with GluR2 were simulated. Our results show that PHF, HMB, and DHMBP formed a partial hydrogen bond with GluR2 in its ligand-binding domain. However, cerbinal was not stable in the ligand-binding domain of GluR2 and induced a significant change in the structure of GluR2. Three-dimensional plots represent the contact and movement situation of the traditional Chinese medicine molecules in the ligand-binding domain. The combined results of the docking and molecular dynamics simulations provide insight into the interaction between these traditional Chinese medicine molecules and proteins.

## 1. Introduction

Traditional Chinese medicine (TCM) plays an important role in Asia, with an increasing number of TCMs being used to treat diseases, such as influenza [1], diabetes [2,3], cancer [4,5,6,7], and neurological disorders [8,9]. TCM has a broad range of applications because it encompasses a large number of compounds. Kum et al. used Jia Wei Liu Jun Zi Tang to treat Parkinson’s disease in clinical trials and improve patients’ communication abilities [8]. Hung et al. investigated the inhibitory effect of a series of TCMs on human histone deacetylase 2, which is implicated in Alzheimer’s disease [9]. These studies show that TCM have beneficial effects in neurological disorders.

In neurological disorders, the α-amino-3-hydroxy-5-methyl-4-isoxazolepropionic acid (AMPA) receptor plays a key role in synaptic plasticity. The AMPA receptor is one of the ionotropic glutamate receptors of ligand-gated ion channels [10,11]. Ionotropic glutamate receptors (iGluRs) mediate the majority of excitatory neurotransmission in the brain [12] and play important roles in neuronal development [13]. iGluRs are membrane-bound receptor ion channels composed of four subunits surrounding a central ion channel, each contributing to pore formation. The AMPA receptors are composed of four types of glutamate receptor subunits (GluR1, GluR2, GluR3 and GluR4) and have a high affinity for full agonists and a low affinity for partial agonists [14,15]. In particular, the structure of the AMPA subunit GluR2, when bound to a wide variety of agonists, partial agonists, and antagonists, has provided compelling clues for the structural basis of channel activation and desensitization [16].

Crystallographic studies have revealed that full and partial agonists bind to the cleft of the GluR2 ligand-binding domain [17]. The ligand-binding domain consists of two weakly interacting lobes (S1S2) with an agonist-binding site between the lobes. The binding of an antagonist in the ligand-binding domain decreases the activity of the protein. The conformation of GluR2 was found to vary upon the binding of different antagonists. The binding cleft can be closed [18,19], unchanged [14,20,21], or opened [22], depending on the type of antagonist. When an antagonist binds to the ligand-binding domain of GluR2, the receptor becomes unresponsive to agonists.

In this study, we investigated the effects of a particular drug, WenQingYin, on GluR2. WenQingYin has proven sedative, anti-allergic, and antipyretic properties [23,24,25,26,27] and consists of eight kinds of TCM (*Angelica sinensis*, White Peony Root, *Rehmannia glutinosa*, Szechwan Lovage Rhizome, *Rhizoma coptidis*, *Scutellaria baicalensis*, Amur Corktree Bark, and *Gardenia jasminoides*). According to the database of Traditional Chinese Medicine Integrated Database (TCMID) [28] and TCM Database@Taiwan [29], these TCMs are composed of various different molecules, such as benzene derivatives, psoralen derivatives, flavonoid derivatives, and cerbinal derivatives. Although the target protein has relevant experimental effects on these derivatives, the interactions of these TCM molecules with the target protein are still not clear. We believe that their component molecules may interact with GluR2 and have the desired efficacy.

Computer simulation methods have been widely applied in studying various systems [30,31,32,33], especially the process of protein folding/unfolding [34], protein docking [35], and protein conformation [36]. Additionally, molecular dynamics (MD) simulation is rapidly becoming a standard tool in studying the structure and dynamics of proteins. Combining docking and molecular dynamics simulations are also used in design and develop inhibitors [37,38]. TCMs are a complex mixture and it is difficult to distinguish the effective component molecules. In the current study, MD simulations were carried out to investigate the interactions between the individual component molecules in WenQingYin and the protein, GluR2. This work can assist in improving our understanding of the mechanisms underlying TCM’s effects and to distinguish the characteristics of individual molecules in TCM.

## 2. Results and Discussion

**Figure 1:**
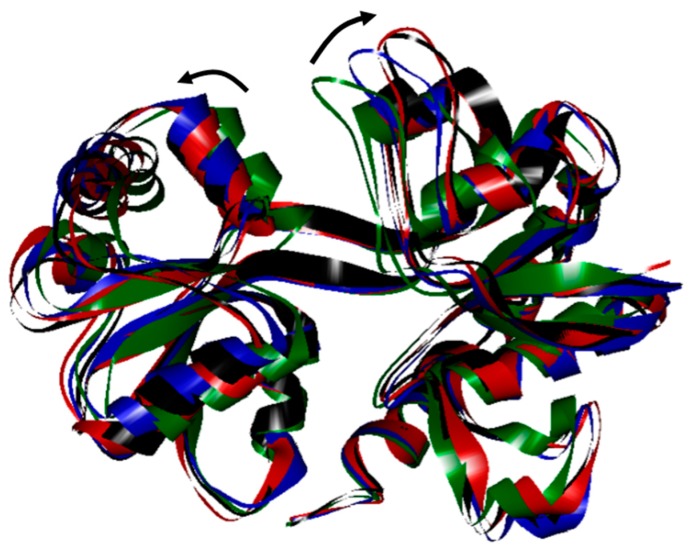
Comparison of structural changes of glutamate receptor 2 (GluR2) during simulation. The green line indicates the crystal structure of GluR2. The blue and red lines represent the structure at 0.4 and 1.2 ns, respectively. The black line represents the final structure of GluR2 at 3.0 ns. The arrows represent the direction of structural change.

To obtain a more realistic structure for GluR2 in aqueous solution, the water molecules were placed in the ligand-binding domain of GluR2 and the system was simulated for 3.0 ns. The crystal structure of GluR2 underwent structural changes to form the relaxation structure during simulation (Figure 1). The structure of GluR2 changed significantly at 0.4 ns and opened continuously at 1.2 ns. Finally, GluR2 reached a thermal equilibrium at 3.0 ns. This figure shows that, after MD simulation, the active zone of GluR2 in aqueous solution becomes larger than that before the water molecules were added. In addition, the structure of GluR2 becomes more flexible and loose, which helps a drug to enter more freely into the ligand-binding domain. The widened cleft of GluR2 allows the entrance of large molecules; hence, the GluR2 protein is more active.

Traditional Chinese medicine contains a wide variety of molecules, which can differ greatly in molecular structure, chemical nature, and size. To accommodate large molecules, the protein must have a less-packed structure that allows the drug molecules to enter. The larger cleft of GluR2 allows drugs to access its active zone more easily and thereby reveal any interactions with the protein. In our simulation, the less-packed structure of GluR2 in water obtained from the MD simulations was used to investigate the docking of the TCM molecules.

**Figure 2:**
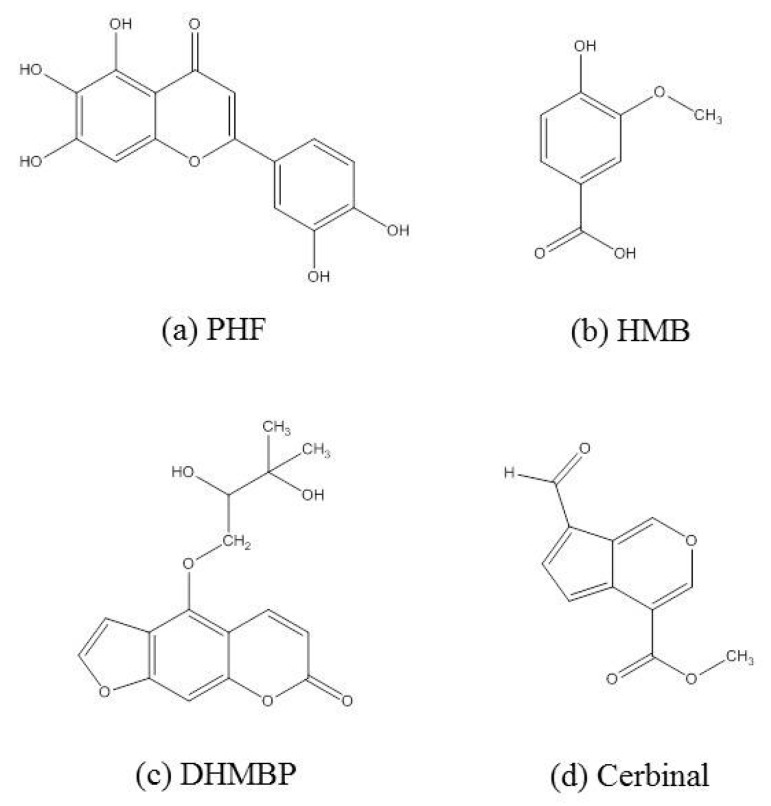
The structures of (**a**) 2-(3,4-dihydroxyphenyl)-5,6,7-trihydroxy-4*H*-chromen-4-one (PHF); (**b**) 4-hydroxy-3-methoxybenzoic acid (HMB); (**c**) 4-(2,3-dihydroxy-3-methylbutoxy)-7*H*-furo[3,2-g]chromen-7-one (DHMBP); and (**d**) methyl 7-formylcyclopenta[*c*]pyran-4-carboxylate (Cerbinal) molecules in WenQingYin.

According to the different structure of benzene, psoralen, flavonoid and cerbinal derivatives, the selected TCM molecules included 2-(3,4-dihydroxyphenyl)-5,6,7-trihydroxy-4*H*-chromen-4-one (PHF), 4-hydroxy-3-methoxybenzoic acid (HMB), 4-(2,3-dihydroxy-3-methylbutoxy)-7*H*-furo[3,2-g]chromen-7-one (DHMBP), and methyl 7-formylcyclopenta[*c*]pyran-4-carboxylate (cerbinal). The structures of these drugs are shown in Figure 2.

**Figure 3:**
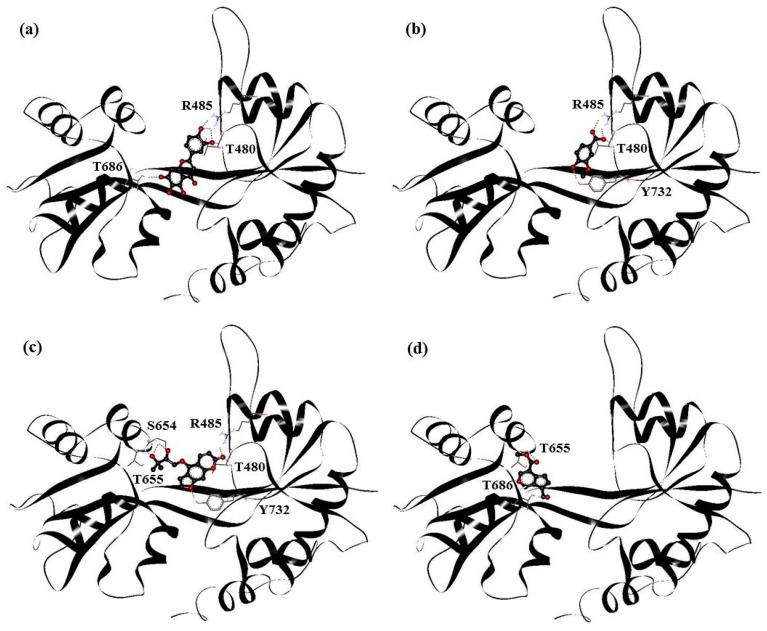
The docking result of (**a**) PHF; (**b**) HMB; (**c**) DHMBP; (**d**) cerbinal system. The red and black balls represent oxygen and carbon atoms. The black line represents backbone of GluR2.

PHF, HMB, DHMBP and cerbinal represent the different types of molecules in WenQingYin. These four molecules were selected for docking into GluR2 and the subsequent MD simulations. Figure 3 shows the complex structures of these molecules and GluR2. The structures were obtained using AutoDock 4.2 software (The Scripps Research Institute, La Jolla, CA, USA), by starting with the drugs placed in the cleft separating the S1 and S2 domains. The figure shows that PHF was in the S1 domain, HMB and cerbinal were in the S2 domain, and DHMBP was in the middle of the ligand-binding core and formed hydrogen bonds with residues in the S1 and S2 domains.

The docking results show the energy-optimized structures of the drugs in the active zone of GluR2. The stabilities of the complexes are mainly attributable to hydrogen bonding. Our results showed that PHF forms hydrogen bonds with T480, R485 and T686; HMB with T480, R485 and Y732; DHMBP with T480, R485, S654, T655 and Y732; cerbinal with T655 and T686. All of these drugs form partial hydrogen bonds, which is in agreement with the crystal structure of the GluR2-glutamate or GluR2-AMPA complex [14]. This is consistent with previously reported mechanisms of GluR2 antagonists [15]. These traditional Chinese medicines can be classified as antagonists of GluR2.

Table 1 shows the binding energies of these drugs. According to experimental results, the protein-ligand binding energy of GluR2 in wild-type protein with glutamate is −8.62 kcal/mol [39]. The binding energies obtained from these docking simulations are all slightly higher than the experimental values, because the simulated GluR2 protein adopted a less-packed structure. The differences in binding energy between our various TCM molecules and GluR2 are extremely small and insignificant. MD simulations were undertaken next to provide further insights into the stability of the drugs in the ligand-binding domain.

**Figure 4:**
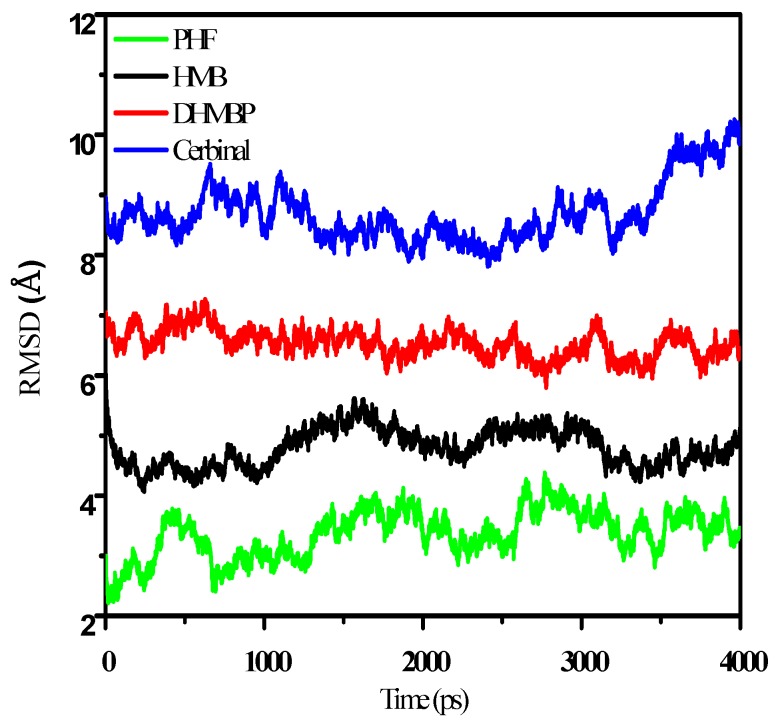
The root mean square displacement/deviation (RMSD) of PHF, HMB, DHMBP and cerbinal system. The curve was separated in parallel for easy viewing.

To study the behavior of the GluR2 ligand-binding domain in the presence of different drugs, a 4.0 ns MD simulation was carried out for each of these complexes. The structural changes of the protein in the drug–protein complex can be investigated by the calculation of root mean square displacement/deviation (RMSD),

(1) $$\mathrm{RMSD}=\sqrt{\langle {\left|\vec{R}\left(t\right)-\vec{R}\left(0\right)\right|}^{2}\rangle }$$

where $\vec{R}$ is the position vector of the atom and the bracket denotes long time-average. A larger RMSD value indicates a lager change in the structure. Figure 4 shows the simulated RMSDs of GluR2 in the drug–protein complexes. For easy viewing, we translated and separated the curves to observe the changes in RMSD throughout the simulation time. In Figure 4, the RMSD values of the GluR2 structure reached equilibrium in the PHF, HMB and DHMBP systems and exhibited significant change after 3.5 ns in the cerbinal system. This indicates significant changes in protein structures when binding with the cerbinal molecule.

**Figure 5:**
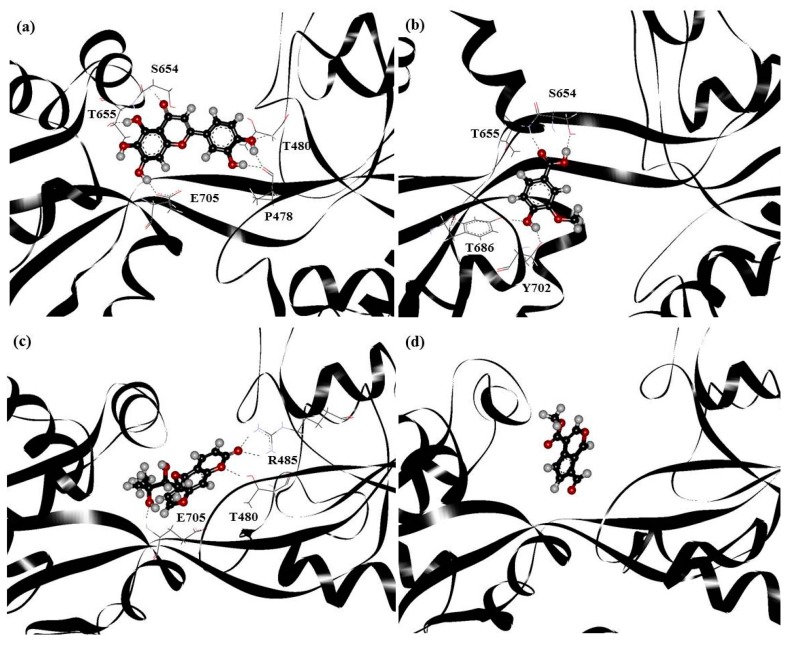
The snapshot of molecular dynamics simulation in (**a**) PHF; (**b**) HMB; (**c**) DHMBP; (**d**) cerbinal systems. The red, black and grey balls represent oxygen, carbon and hydrogen atoms. The black line represents backbone of GluR2.

Since all the selected drugs have polar groups, hydrogen bonding between these polar groups and the polar residues of the protein are the dominant interactions. Figure 5 shows snapshots of the configurations of drug–protein complexes obtained from equilibrated MD trajectories. The figure shows that PHF formed hydrogen bonds with residues S654, T655, T686 and Y702; HMB formed hydrogen bonds with residues R485 and E705; DHMBP formed hydrogen bonds with residues S654, T655, E705, P478 and T480. However, no hydrogen bonds were formed between the cerbinal molecule and GluR2 protein.

Our results show that the PHF, HMB and DHMBP molecules interacted with GluR2 more strongly than the cerbinal molecule. MD simulations show that the PHF, HMB and DHMBP molecules formed hydrogen bonds with residues P478, T480, R485, S654, T655, T686, Y702 and E705 of GluR2. These results are somewhat different from those obtained in the docking process (in Figure 3). In the docking investigation, in addition to the residues mentioned above, we found that the molecules also formed hydrogen bonds with residues T480, R485, S654, T655, T686 and Y732. Since the drug–protein complex was simulated under aqueous conditions using MD, its results should be more reliable than those of the docking simulation. The agonistic ligands glutamate and AMPA formed hydrogen bonds with residues P478, T480, R485, S654, T655 and E705 in the GluR2 ligand-binding domain [14]. The active site of GluR2 is in the vicinity of these amino acids. Our results suggest that antagonists form partial hydrogen bonds with GluR2 that affect the binding of agonists with the GluR2 protein. For example, 6,7-dinitroquinoxaline-2,3-dione (DNQX) has been shown to form hydrogen bonds with residues Y450, T480, R485, T686 and Y732 when bound to GluR2 [15]. From the simulation, we may infer that, because PHF, HMB and DHMBP formed partial hydrogen bonds with GluR2, these molecules are therefore possible antagonists of GluR2. However, because cerbinal did not form any hydrogen bonds with GluR2, its effect on the protein should differ from that of the other molecules, and it may therefore not be an antagonist for GluR2.

The hydrogen bonds of the drug–protein complexes also affect the conformation of GluR2. The pairs of residues (Y732 and E705) and (E402 and T686) in GluR2 formed intramolecular hydrogen bonds, leading to the conformational change of the protein from an open to a closed state [12,40,41]. The interactions between these residues are the major factors in the cleft-closing process of GluR2. Our simulation shows that PHF, HMB and DHMBP formed hydrogen bonds with these residues (PHF with E705; HMB with T686; DHMBP with E705) and hence prevented the cleft-closing process of GluR2. The configuration of the GluR2 protein did not change significantly in these drug–protein systems, as shown in Figure 4, and the protein maintained its less-packed structure during each simulation.

**Figure 6:**
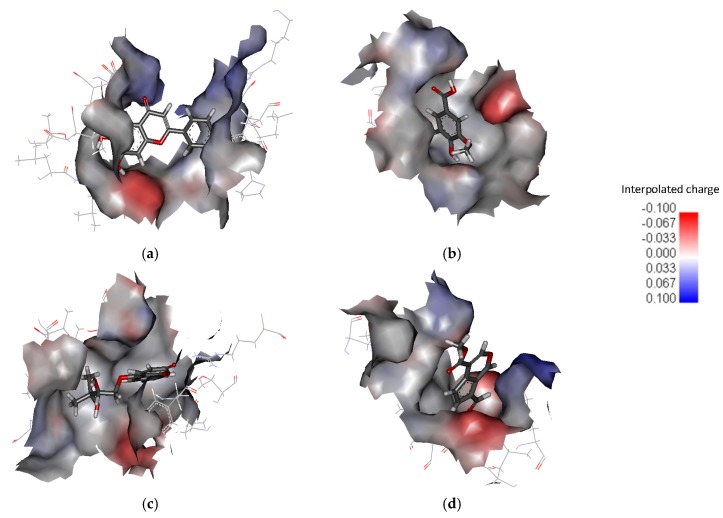
Interpolated charge surfaces around (**a**) PHF; (**b**) HMB; (**c**) DHMBP; and (**d**) cerbinal molecule.

The interpolated charge surfaces around the drugs are shown in Figure 6. In addition to the apparent electrostatic force, the drug is encircled by hydrophobic interactions in Figure 6a–c. PHF, HMB and DHMBP are trapped in the ligand-binding domain. In Figure 6d, there is a noticeable opening next to the cerbinal molecule. The residues around the drug are list in Table 2. PHF, HMB, and DHMBP molecules are surrounded by more hydrophilic and hydrophobic residues. The interaction between these drugs and GluR2 is stronger than that between cerbinal and GluR2.

To explore the interaction between drugs and proteins further, the MD trajectories were analyzed and various radial distribution functions (RDFs) were calculated. RDFs between different atoms *i* and *j* are calculated as follows [30]:

(2) $$g_{ij}\left(r\right)=\frac{V\langle ∆N_{ij}\left(r\to r+∆r\right)\rangle }{4\pi r^{2}∆rN_{i}N_{j}}$$

where $\langle ∆N_{ij}\left(r\to r+∆r\right)\rangle$ is the ensemble averaged number of j around i within a shell from distance *r* to *r* + ∆*r*, *V* is the system volume, and *N_i_* and *N_j_* are the numbers of *i* and *j*, respectively.

**Figure 7:**
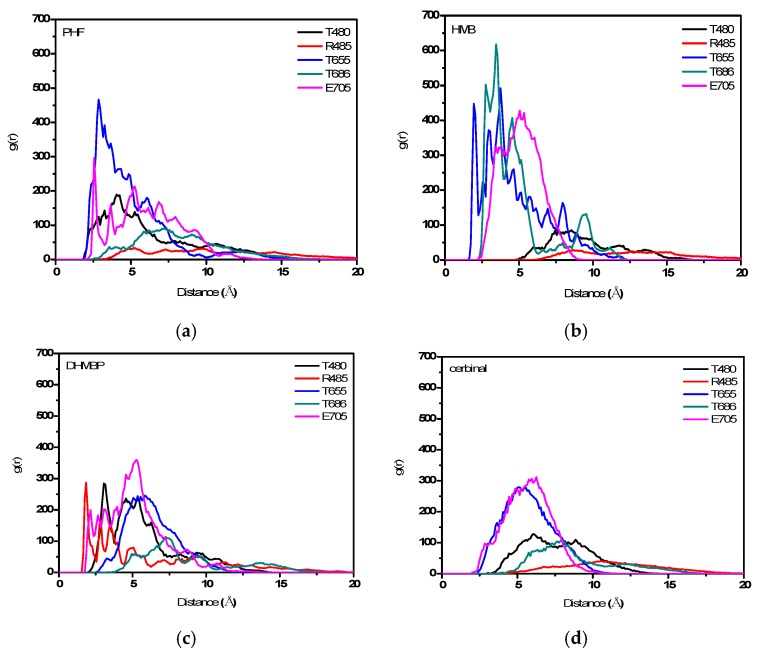
Radial distribution functions (RDFs) of (**a**) PHF; (**b**) HMB; (**c**) DHMBP; and (**d**) cerbinal with different residues. Black line: T480, red line: R485, blue line: T655, dark cyan line: T686, and magenta line: E705.

The plots of *g*(*r*) for the oxygen atoms in drug molecules with different residues are shown in Figure 7. The residues were selected owing to their association with the conformational changes in GluR2 as well as the effect of antagonists. Figure 7a shows that PHF exhibits major peaks, which appear at approximately 2.55 and 2.85 Å with E705 and T655, respectively. Figure 7b shows that HMB has major peaks, which appear at approximately 1.95 and 2.75 Å with T655 and T686, respectively. In addition, HMB shows a broad peak at around 5 Å with E705. Figure 7c shows that DHMBP has major peaks, which appear at approximately 1.85, 2.15 and 3.05 Å with R485, E705 and T480, respectively. Figure 7d shows that cerbinal has broad peaks at around 5 Å with T655 and E705.

RDFs provide an insight into the intermolecular interactions during the simulation process. This finding indicates that these drugs have strong interactions with specific residues. The main residues that interacted with PHF are T655 and E705, with HMB are T655 and T686, and with DHMBP are R485, E705 and T480. These residues showed no significant interaction with cerbinal. The results suggest that a large number of intermolecular forces stabilize the interaction between the residues and drugs (PHF, HMB, and DHMBP).

**Figure 8:**
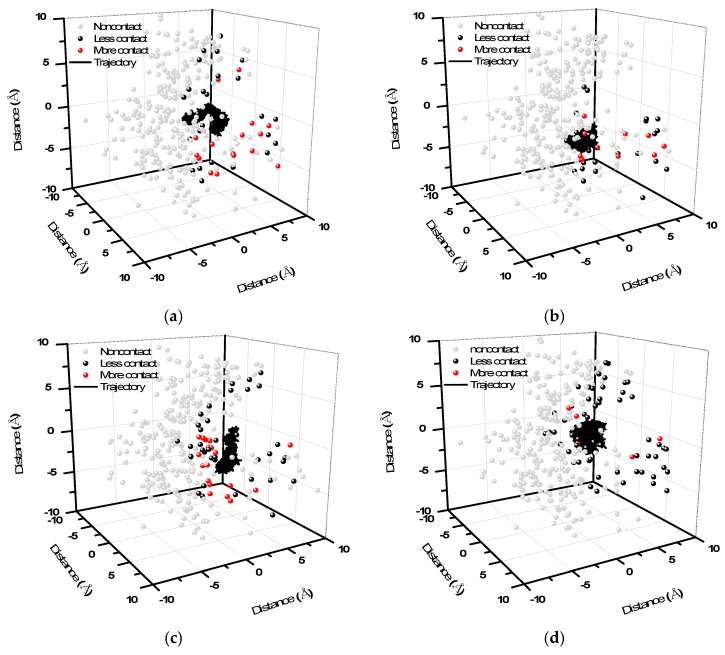
The 3D diagram of contact atoms around the ligand-binding domain in (**a**) PHF; (**b**) HMB; (**c**) DHMBP; (**d**) cerbinal system. The gray dots show the noncontact atoms around the ligand-binding domain. The black dots show the less contact atoms. The red dots show the more contact atoms. The black line shows the ligand trajectory during the simulation time.

Figure 8 presents the simulated trajectories of the drugs in the GluR2 ligand-binding domain. These depictions help in understanding the movement and situation of drugs in contact with the surrounding environment. In Figure 8, the simulated trajectories of the center of mass (CM) of the drugs in the ligand-binding domain are shown. The drug-atom distance is the distance between the CM of the drug and its neighboring atoms in the protein. We defined “contact” as when the drug-atom distance was less than 10 Å. The description “less contact” is used when there are more than 100 instances of contact; “more contact” is used with more than 3000 instances of contact. The figures show the contact frequencies of the protein atoms with the drugs.

**Figure 9:**
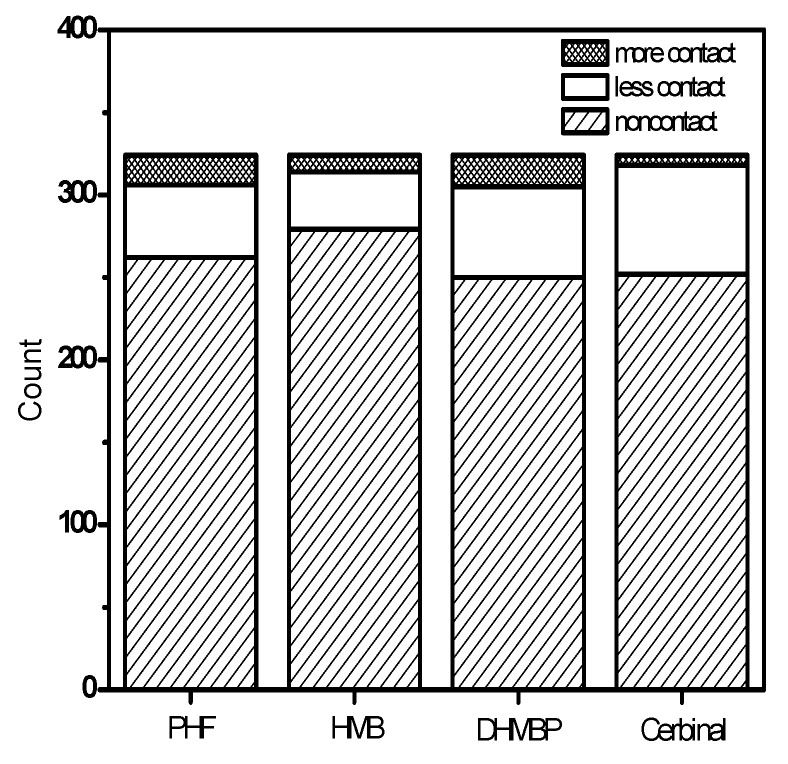
All statistical distribution of atoms in the frequency of contact at PHF, HMB, DHMBP and cerbinal systems.

In Figure 8a–c, the trajectories of the drugs are confined to a small zone, because these drugs interact strongly with GluR2 in the active zone and form hydrogen bonds. The figures show that the (**a**) PHF, (**b**) HMB and (**c**) DHMBP molecules occupy the ligand-binding domain. Figure 8d shows that the trajectories of the cerbinal molecule occupy a larger area than the other drugs in the ligand-binding domain. This indicates that the drug moves more freely in the ligand-binding domain, because no hydrogen bonds are formed with GluR2. The figure also shows that the frequencies of contact are different in these systems. In Figure 8, the gray dots show the atoms located on the protein within the ligand-binding domain that do not experience contact; the black dots show the atoms with low contact frequency; and the red dots show the atoms with high contact frequency. Figure 9 shows the distribution of atoms by contact type (no contact, low frequency, and high frequency) in each system, and it can be seen that the distributions differ between the systems. There are fewer atoms with high contact frequency in the cerbinal system than in the PHF, HMB and DHMBP systems. This is consistent with the previous observation that cerbinal has no clear intermolecular interaction with the protein and hence moves more freely in the ligand-binding domain. In contrast, PHF, HMB and DHMBP form hydrogen bonds with specific residues, causing the molecules to come into contact with GluR2 atoms more frequently.

**Figure 10:**
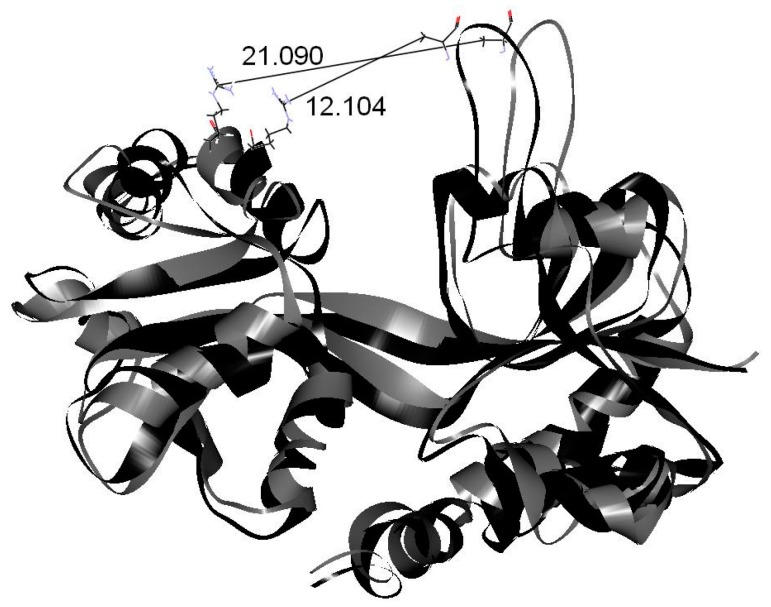
The backbone of GluR2 in cerbinal system at 2.0 ns and 4.0 ns. The black line is 2.0 ns and the gray line is 4.0 ns. The distance between A455 and R660 is shown.

Cerbinal is one of the main components of WenQingYin. From our simulations, we found that, unlike the other three compounds (PHF, HMB and DHMBP), cerbinal does not interact obviously with GluR2. Additionally, the RMSD value for the cerbinal system changes significantly between 2.0 ns and 4.0 ns, as shown in Figure 4. Figure 10 shows the configurations of the backbone of GluR2 in the cerbinal–protein system at the simulation times 2.0 ns and 4.0 ns. The calculated distance between residues A455 and R660, located on opposing sides of the cleft [12], is 12.104 Å at 2.0 ns and 21.090 Å at 4.0 ns, as shown in the figure. This result indicates that the cleft of the ligand-binding domain of cerbinal–GluR2 opens readily and allows the drug to leave easily. Based on this, we infer that cerbinal is not an antagonist for GluR2 in WenQingYin.

According to TCMID [28], there are a series of related drugs that have experimental and effective data associated with GluR2. Previous studies utilized a computational approach to determine the interactions of drugs such as DNQX, guanosine monophosphate (GMP), willardiine, and kainite with GluR2 [15,42,43]. These components bind with GluR2 in the ligand-binding domain and inhibit its glutamatergic activity. Similar hydrogen bonds were formed with GluR2 in the PHF, HMB and DHMBP system. Competitive antagonism of GluR2 requires a ligand that makes canonical binding interactions with the ligand-binding domain [15]. The binding of PHF, HMB and DHMBP to the ligand-binding domain appear to result in stable complexes. PHF, HMB and DHMBP are components of WenQingYin that affect GluR2.

## 3. Methods

The S1S2 domain of GluR2 of the AMPA receptor was modeled on the protein structure adopted from the crystal structure with the PDB code 1M5B. The structures of selected TCM molecules (PHF, HMB, DHMBP and cerbinal) are shown in Figure 2.

MD simulations were performed using the PMEMD (particle mesh ewald molecular dynamics) module of AMBER12 (University of California, San Francisco, CA, USA). To simulate the protein in the aqueous solution, adequate water molecules were placed into the active site. The system was first simulated for 3.0 ns to reach thermal equilibrium. In this situation, the protein became more flexible, which is more realistic. After the 3.0 ns simulation, we found that the structure of the active zone of GluR2 had opened more widely. This protein structure was used as the starting point for the subsequent docking and MD simulations.

Molecular dockings of the four selected ligand molecules to pre-MD-simulated GluR2 was performed with AutoDock 4.2 software (The Scripps Research Institute). The program allows the ligand to achieve full flexibility, while keeping the geometry of the receptor frozen. The docking procedure was restricted to the cleft between the two subdomains that make up the ligand-binding domain. An active site of 15 Å around the ligand-binding domain was created. The exploration of docking positions was carried out using 100 runs of the Lamarckian genetic algorithm, using the AutoDock default parameters.

In the docking procedures, an adequate number of Cl^−^ ions was added into the system to neutralize the charge of the system. After docking, the protein-ligand complex was immersed in a rectangular solvent box, maintaining a distance of 8 Å between the wall of the box and the closest atom of the solute. The counterions and water solvent molecules were added using the LEaP (link, edit, and parm) module of AMBER12 (University of California). The TIP3P model of water was chosen for the MD runs. Initial relaxation of each complex was achieved by performing 10,000 steps of energy minimization using a cutoff of 10.0 Å. These optimized complex structures were used to start the subsequent MD simulations. The AMBER ff99SB force field was selected for these simulations. In all of the simulations, the temperature was set to 300 K and periodic boundary conditions were applied. For each system, the production run of 4.0 ns was performed.

## 4. Conclusions

We investigated the effects of four traditional Chinese medicine molecules (PHF, HMB, DHMBP and cerbinal), which are the main compounds in WenQingYin, upon the AMPA receptor by using MD simulations. In summary, PHF, HMB and DHMBP formed partial hydrogen bonds with GluR2 in the ligand-binding domain, which led to the formation of more stable drug–protein complexes, and these molecules are antagonists for GluR2. However, cerbinal did not form hydrogen bonds with GluR2, and the cleft of the ligand-binding domain opened during cerbinal’s simulated interaction with GluR2. THF, HMB and DHMBP were trapped in the ligand-binding domain by hydrophilic and hydrophobic residues. Three-dimensional plots representing the frequency with which these drugs made contact with GluR2 show that PHF, HMB and DHMBP bound specific atoms and the cerbinal molecule moved more freely in the ligand-binding domain. Based on the results of simulations, we suggest that computer simulation is a powerful approach to explore the interaction between various components of traditional Chinese medicine and a specific protein. These results can be useful in understanding the development of traditional Chinese medicine molecules in neurological disorders further.

## Figures and Tables

**Table 1:** The binding energy of drugs in docking.

Drugs	Binding Energy (kcal/mol)
2-(3,4-dihydroxyphenyl)-5,6,7-trihydroxy-4*H*-chromen-4-one (PHF)	−7.69
4-hydroxy-3-methoxybenzoic acid (HMB)	−5.26
4-(2,3-dihydroxy-3-methylbutoxy)-7*H*-furo[3,2-g]chromen-7-one (DHMBP)	−6.57
Cerbinal	−5.53

**Table 2:** Residues around the drugs.

Drugs	Residues
PHF	Y450, P478, L479, T480, R485, T649, L650, G653, S654, T655, L703, L704, E705, Y732
HMB	T649, L650, G653, S654, T655, T686, Y702, L703, L704, E705, M708
DHMBP	Y450, T480, R485, L498, G499, I500, T649, L650, S654, T655, T686, Y702, L703, L704, E705, S706
Cerbinal	L498, G499, I500, L650, S654, T655, E705, D728, K730
